# Strengthening primary care with the world health organization’s basic package of interventions for rehabilitation: developing a list of interventions and a clinical resource

**DOI:** 10.1186/s12875-026-03479-z

**Published:** 2026-10-16

**Authors:** Abena Tannor, Dima Touhami, Antony Duttine, Alexandra Rauch, Pauline Kleinitz, Elanie Marks, Alarcos Cieza, Carla Sabariego

**Affiliations:** 1 Faculty of Health Sciences and Medicine, University of Lucerne, 6002 Lucerne, Switzerland; 2 Department of Noncommunicable Diseases and Mental Health, World Health Organization, Geneva, Switzerland; 3 Center for Rehabilitation in Global Health Systems, WHO Collaborating Center, Faculty of Health Sciences and Medicine, University of Lucerne, 6002 Lucerne, Switzerland; 4 Swiss Paraplegic Research, Nottwil, Switzerland

**Keywords:** Primary care, Rehabilitation, Functioning, Task-sharing

## Abstract

**Background:**

Access to rehabilitation, a critical component of universal health coverage (UHC) remains limited, particularly in primary care (PC) and low-resource settings. To address this gap, the WHO Rehabilitation Programme developed the Basic Package of Interventions for Rehabilitation (Basic PIR). This toolkit is designed to empower PC non-rehabilitation workers to deliver a prioritised set of easy, safe and effective rehabilitation interventions through task-sharing. This article describes the participatory process used to develop the WHO's Basic PIR, specifically its list of interventions and guiding clinical resource.

**Methods:**

The development of the Basic PIR utilized a three-phase participatory methodology. In Phase 1, interventions were extracted from the WHO’s Package of Interventions for Rehabilitation (PIR) using feasibility criteria and prioritised via a two-stage modified Delphi process with an international Technical Working Group. Phase 2 structured these interventions into clinical targets by adapting existing WHO task-sharing frameworks and clinical practice guidelines. In Phase 3, the prototype resource was field-tested during 5-day training sessions with PC workers in Ghana and Fiji, using qualitative group discussions to evaluate usability and guide final structural revisions. This participatory process was coordinated by a five-member WHO Rehabilitation Programme internal review panel to optimize the tool for PC and low-resource settings.

**Results:**

In Phase 1, thirteen functioning domains related to health conditions were identified, leading to the preselection of 188 out of 297 interventions for rehabilitation from the WHO’s Package of Interventions for Rehabilitation, with 71 interventions ultimately included in the Basic PIR. In Phase 2, a prototype clinical resource with eight functioning domains and corresponding intervention targets was created. The preliminary field tests in Phase 3 resulted in specific modifications, including the revision of rehabilitation steps and the development of new patient handouts to improve usability and clarity.

**Conclusion:**

By promoting a functioning-based approach, the Basic PIR aims to improve access to rehabilitation services in primary care and low-resource settings, which has the potential to contribute to the achievement of UHC. Future pilot studies will evaluate its feasibility and acceptability, guiding its broader implementation.

**Supplementary Information:**

The online version contains supplementary material available at https://doi.org/10.1186/s12875-026-03479-z .

## Background

Primary health care is a whole-of-society approach to effectively organize and strengthen national health systems to bring services for health and wellbeing closer to communities [1]. Primary Health Care is essential for achieving universal health coverage (UHC) and meeting health-related sustainable development goals, especially in resource-limited areas [2, 3]. It can provide 90% of the necessary interventions for UHC to deliver comprehensive health services directly to communities [4]. Five essential health services—health promotion, prevention, treatment, rehabilitation, and palliative care—have been recognized by the Alma-Ata and Astana declarations as key components of primary health care [5, 6]. Primary care (PC), a key component of primary health care, is a model of care that supports first-contact, accessible, continuous, comprehensive, and coordinated person-focused care [7]. Access to rehabilitation is vital in the health care continuum and is equally important in PC settings as it is in secondary or tertiary care [8, 9].

The World Health Organization (WHO) defines rehabilitation as “a set of interventions designed to optimize functioning and reduce disability in individuals with health conditions in interaction with their environment” [10]. Therefore, rehabilitation plays a vital role in optimizing functioning in daily life, enhancing well-being [11] and contributing to improving the effectiveness of other health services, such as prevention and palliative care [12, 13]. As stressed in a recently passed World Health Assembly resolution requesting that rehabilitation be strengthened in health systems, rehabilitation is relevant for individuals of all age groups, with various acute or chronic illnesses, and at all stages and levels of care [14].

The need for rehabilitation is increasing globally. Current estimates suggest that approximately 2.6 billion individuals could benefit from rehabilitation for diverse health conditions at some point of their lives [15]. The increase is driven by factors including aging populations, an increase in injuries, the high prevalence of noncommunicable diseases (NCDs), such as heart disease, diabetes, cancer and musculoskeletal conditions as well as the long-term consequences of communicable diseases like HIV and COVID-19 [16, 17]. Additionally, advances in curative care have led to more people surviving illnesses and injuries, many of whom now require rehabilitation to optimize their functioning [18–21]. Despite the global need, rehabilitation services are often inadequately integrated into PC settings, particularly in low- to middle-income countries (LMICs), making access to rehabilitation services a significant challenge [20, 22–25]. Low access rates can have a detrimental effect on health outcomes for individuals with rehabilitation needs [22, 26]. Key barriers to access rehabilitation services include a lack of clear evidence-based guidance on interventions and service models specifically tailored for PC settings [27]. Other barriers include poor governance and leadership, insufficient funding and resources, ineffective referral networks, urban-centric service concentration, transport barriers and a shortage of skilled personnel [20, 28–30]. This shortage is particularly acute in PC and low-resource settings, with fewer than 10 skilled rehabilitation practitioners per million people [31, 32]. To expand the coverage and accessibility of rehabilitation services in these situations, innovative approaches are necessary.

Several models can support the integration of rehabilitation services into PC settings, including clinic-based, outreach, tele-rehabilitation, self-management support, shared care, community-based and case management [27, 33–35]. These models underscore the importance of a collaborative multidisciplinary team approach, interprofessional trust, leadership, communication, and clearly defined roles for successful integration [33, 35]. Common rehabilitation interventions in PC settings, such as functioning assessments, therapeutic exercises, psychological interventions, and self-management education, as well as their coordination, are essential components of all models [34].

A key strategy for integrating rehabilitation in PC settings, especially where there are few or no professionals trained in rehabilitation such as doctors and nurses, is task sharing [36, 37]. Task sharing expands the delivery of health services to lay and mid-level healthcare professionals rather than taking tasks away from higher-level providers, which helps address provider shortages in many settings [38]. There is, however, the need to outline the role of PC non-rehabilitation workers (defined as those not trained in rehabilitation), such as doctors and nurses, in the provision of rehabilitation through task sharing [25, 37]. This should be supported by the establishment of good referral systems. Indeed, recent work has stressed the key role of nurses in delivering rehabilitation interventions via PC settings [39]. Sharing rehabilitation tasks with nonprofessional community workers, informal caregivers or peer supporters could be another strategy in low-resource settings. However, evidence on the effectiveness of this approach is inconclusive [40].

To improve access to rehabilitation in PC with low to no availability of rehabilitation specialists such as physical and rehabilitation physicians, physical therapists, occupational therapists, rehabilitation nurses and speech and language therapists, the WHO has developed the Basic Package of Interventions for Rehabilitation (Basic PIR), a toolkit for primary care non-rehabilitation workers. The Basic PIR comprises three components - a list of interventions, a clinical resource, and an implementation guide. It is designed to empower a specific group: PC non-rehabilitation workers enabling them to provide essential, basic rehabilitation services. It enables the delivery of a carefully selected set of prioritised, safe, and easy-to-learn evidence-based interventions, with guidance on integrating them into PC settings [41]. In the Basic PIR, clinical decision-making is facilitated through a user-friendly decision-tree structure, similar to existing WHO resources such as the WHO Integrated Care for Older People (ICOPE) [42] and the WHO Mental Health Gap Action Programme for mental, neurological and substance use disorders in nonspecialist health settings (MH-GAP) [43]. Trust in and uptake of any new tool is conditional on a sound and participatory development process. The objective of this article is to describe the participatory development of the WHO’s Basic PIR, and to present the resulting list of interventions and the clinical resource that guides their application.

## Methods

**Fig 1:**
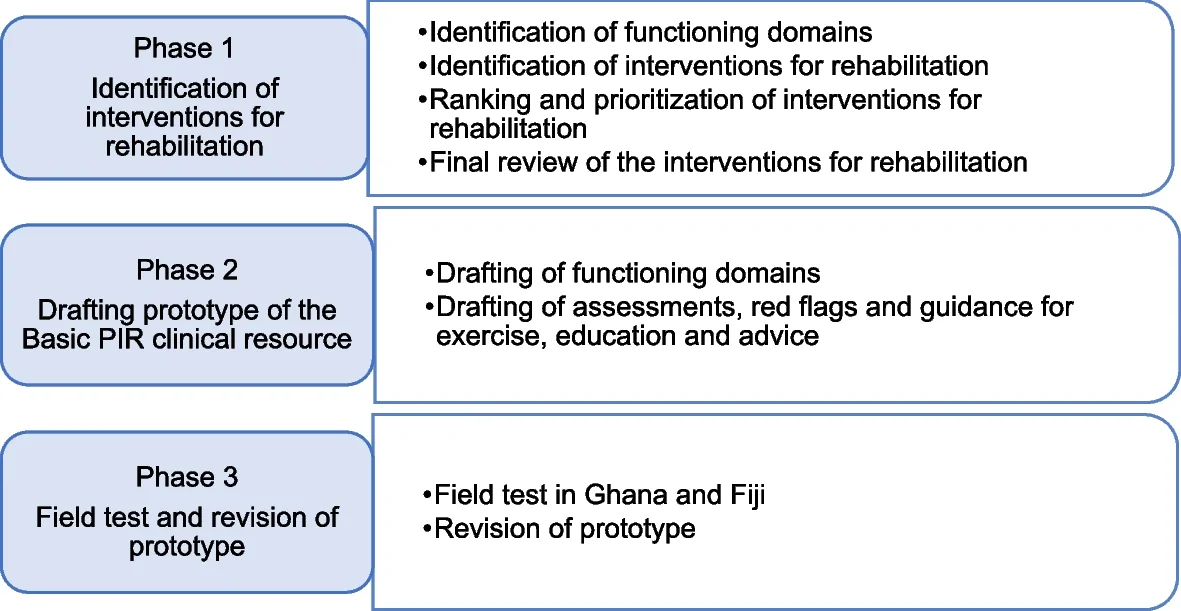
Three-phase methodology used in the development of the Basic Package of Interventions for Rehabilitation (list of interventions and clinical resource)

The development of the Basic PIR list of interventions and clinical resource employed a three-phase methodology (Fig. 1). The initial phase focused on identifying interventions for rehabilitation in PC settings and laid the groundwork for Phase 2, where the core structure and content of the Basic PIR clinical resource were defined. Phase 3 involved conducting preliminary field tests in selected countries to test the content of the prototype Basic PIR clinical resource and to inform corresponding revisions. This process ensured that the Basic PIR was well suited for use in PC settings, particularly in low-resource environments. Throughout the process, a highly participatory approach was implemented, with activities directed and managed by an internal review panel made up of five WHO rehabilitation programme members (hereafter referred to as the internal review panel), established in phase 1. This internal review panel facilitated the feedback and input from a broader and diverse group of experts, ensuring a comprehensive and collaborative development process.

### Phase 1: Identification of interventions for rehabilitation

Phase 1 utilised a participatory, consensus-driven mixed-methods design to systematically extract, evaluate, and prioritise rehabilitation interventions suitable for PC settings. This process included an initial internal screening and mapping phase executed by the internal review panel, followed by an external validation phase utilising a two-stage modified Delphi process with a Technical Working Group (TWG). The five-member internal review panel (four female, one male) included one consultant from the WHO Regional Office for Africa (AFRO), one technical advisor from the WHO Regional Office for the Western Pacific (WPRO), and two consultants alongside one technical advisor from the WHO Regional Office for Europe (EURO). These five experts were selected because of their clinical, programmatic, and health-system perspectives necessary for product development. While all possessed foundational expertise in Rehabilitation and PC, individual members held specialized technical expertise in Health Financing, Workforce Development, Service Provision and Standards, and Health Information Systems. This internal review panel was responsible for the initial drafting, internal advisory, and subsequent iterative review of the toolkit components.

#### Identification of functioning domains

The selection of the Basic PIR's interventions began by identifying functioning domains linked to the 20 health conditions in the WHO's Package of Interventions for Rehabilitation (PIR) (Table 1) [44].

**Table 1:** A summary of the WHO’s Package of Interventions for Rehabilitation (WHO PIR) WHO’s Package of interventions for rehabilitation (PIR)

• Main purpose: Promote access to evidence-based rehabilitation interventions at all service delivery platforms
• Key Components: Essential interventions for 20 health conditions under seven disease areas
• Necessary Resources: Assistive products, equipment, consumables, and workforce
• Uses: Planning and budgeting of rehabilitation services, developing curricula and training materials, and identifying research gaps for defining research agendas
• Target Audience: Ministries of health and other target users, such as service planners, service providers, academics and researchers

^a^PIR: Package of Interventions for Rehabilitation

^a^Disability weights sourced from IHME 2017 data resources: Global Burden of Disease 2017 Disability weights. Accessed here: http://ghdx.healthdata.org/gbd-2017

^a^3 No data was available for this health condition in Global Burden of Disease 2016 or 2017. This health condition were selected by rehabilitation experts during the PIR development process, as they were deemed highly relevant for rehabilitation in low- and middle-income countries

The internal review panel reviewed each disease area addressed in the PIR, aiming to select health conditions on the basis of the following criteria:

- High prevalence in PC settings.
- Coverage of different disease areas (e.g., musculoskeletal, cardiovascular, and neurological conditions).
- Higher global burden of disease disability weights, favouring conditions with greater years lived with disability.
- Higher global prevalence where baseline disability weights are comparable.
- A link to a high number of affected aspects of functioning.

Following a structured review of the epidemiological criteria, the internal review panel reached a formal consensus on the selection of 10 health conditions. Through a subsequent internal online consultation, the internal review panel achieved further consensus to identify and map 13 functioning domains from the PIR that broadly corresponded to these selected conditions. This approach allows the Basic PIR to be applied to a wider range of conditions by focusing on functioning, not just disease.

The WHO International Classification of Functioning, Disability, and Health (ICF) [45] served as a reference framework for categorizing the selected functioning domains into eight overarching areas: (1) mental functions; (2) hearing, speech and communication; (3) vision; (4) swallowing; (5) respiratory and cardiovascular; (6) motor functions and mobility; (7) pain; and (8) self-care and participation.

#### Identification of interventions for rehabilitation

A preliminary list of interventions from the PIR was extracted and reviewed by the internal review panel via an online consultation, based on predetermined criteria. This was a preparatory step for a more extensive participatory process involving a broader group of experts. The predetermined criteria included the following: A suitable intervention:

- can be delivered safely and effectively by PC non-rehabilitation workers based on a protocol and after short online training.
- addresses a functioning problem that is easy to identify by PC non-rehabilitation workers.
- has a duration that is feasible for the non-rehabilitation workers to deliver in PC settings alongside their usual clinical role.
- can be delivered using low-cost or locally available equipment or consumables.

Interventions (*n*=59) for which at least 3 out of the 5 internal review panel members indicated ‘yes’ were included in the preliminary list of preselected interventions. To evaluate and refine the interventions, a 26-member external TWG was established using purposive sampling. Potential candidates were identified through international professional networks, global rehabilitation organizations, and PC societies. To ensure a replicable and balanced panel, candidates had to meet specific eligibility criteria: (1) at least five years of clinical, academic, or policy experience in primary healthcare or rehabilitation; (2) demonstrated professional/clinical expertise in one of the eight designated functioning domains; and (3) a balanced representation across professional backgrounds and geographic regions. Following a formal email invitation and an online screening survey, 26 experts were selected, comprising 17 rehabilitation specialists (including occupational, physical, and speech therapists, orthotists/prosthetists, and rehabilitation physicians) and 9 non-rehabilitation PC professionals (including general practitioners, family physicians, audiologists, ophthalmologists, optometrists, and nurses). This TWG maintained a strict geographic balance, with experts spanning all six official WHO regions.

The TWG used a two-stage modified Delphi process and applied the same criteria established by the internal review panel to conduct this review. Data collection for this first stage was conducted via an online survey where experts provided binary ('yes'/'no') responses regarding intervention suitability. Data analysis involved descriptive frequency statistics to identify consensus which was defined as a minimum of 70% of the TWG members voting 'yes' to retain an intervention. During this selection process, open-ended feedback from the survey allowed the TWG to make recommendations. The internal review panel qualitatively analysed these recommendations and eventually integrated 10 additional interventions that were absent from the initial list but were highly suitable for task-sharing.

#### Ranking and prioritization of interventions for rehabilitation

The internal review panel first compiled the interventions and presented them to the TWG again for ranking and prioritization via an online data collection form, using a 0 (lowest) to 5 (highest) scoring system based on the specified criteria:

1. The benefit achieved through the provision of the intervention in PC settings. The larger the benefit for the person is, the higher the ranking should be. Benefit was defined as either the effect of the intervention on everyday functioning or the reduction in the risk of developing complications and requiring further health interventions.
2. The need for intervention in the PC population. The higher the need is, the higher the ranking should be. Need is defined by the number of people with health conditions who use PC frequently and could benefit from the intervention.
3. The resource requirements for providing interventions in PCs. The lower the requirements are, the higher the ranking. Resource requirements include both the time of the PC non-rehabilitation worker required to deliver the intervention effectively (considering common dosage) and the equipment and materials needed to deliver the intervention.
4. The ease with which PC non-rehabilitation workers can deliver the intervention safely and effectively. The easier the intervention is to deliver, the higher the ranking should be. The ease of providing an intervention correlates with its features, that is, how simple it is, either being of low complexity or containing few components. It also refers to how little individual adaptation and tailoring an intervention requires and to low levels of contraindications.

Second, TWG members were asked to prioritize interventions by selecting the 10 interventions that would have the greatest impact on the health and functioning of their populations.

#### Final review of the interventions for rehabilitation

Following the ranking and prioritization process, the final selection of interventions was determined using a multi-stage consensus analysis integrating both numerical scores and prioritization votes. First, the 0–5 ranking data was evaluated by calculating arithmetic mean scores to establish baseline consensus on clinical feasibility and benefit, with an average score of 3.0 or higher required for an intervention to be considered for inclusion (Criterion 1). Next, these ranked datasets were filtered against a secondary threshold requiring a minimum frequency of prioritization votes from five or more TWG members to identify high-impact interventions (Criterion 2). Interventions meeting both criteria were selected, ensuring equitable clinical representation across all eight functioning domains. Modifications were made to the list, including adding compression garments and urinary catheters, excluding group conversation and soft tissue techniques due to feasibility, and combining modified sports with recreation activities. Additionally, while standalone clinical trial evidence for thermotherapy can be mixed, basic thermotherapy was retained as an independent intervention because it was directly derived from the validated Clinical Practice Guidelines of the parent WHO PIR and was explicitly prioritised by the TWG as a safe, low-cost self-management modality. To resolve remaining discrepancies, these changes were presented during a final web conference, where qualitative feedback was synthesized to secure TWG consensus and final approval.

### Phase 2: Drafting the prototype of the Basic PIR clinical resource

**Fig. 2:**
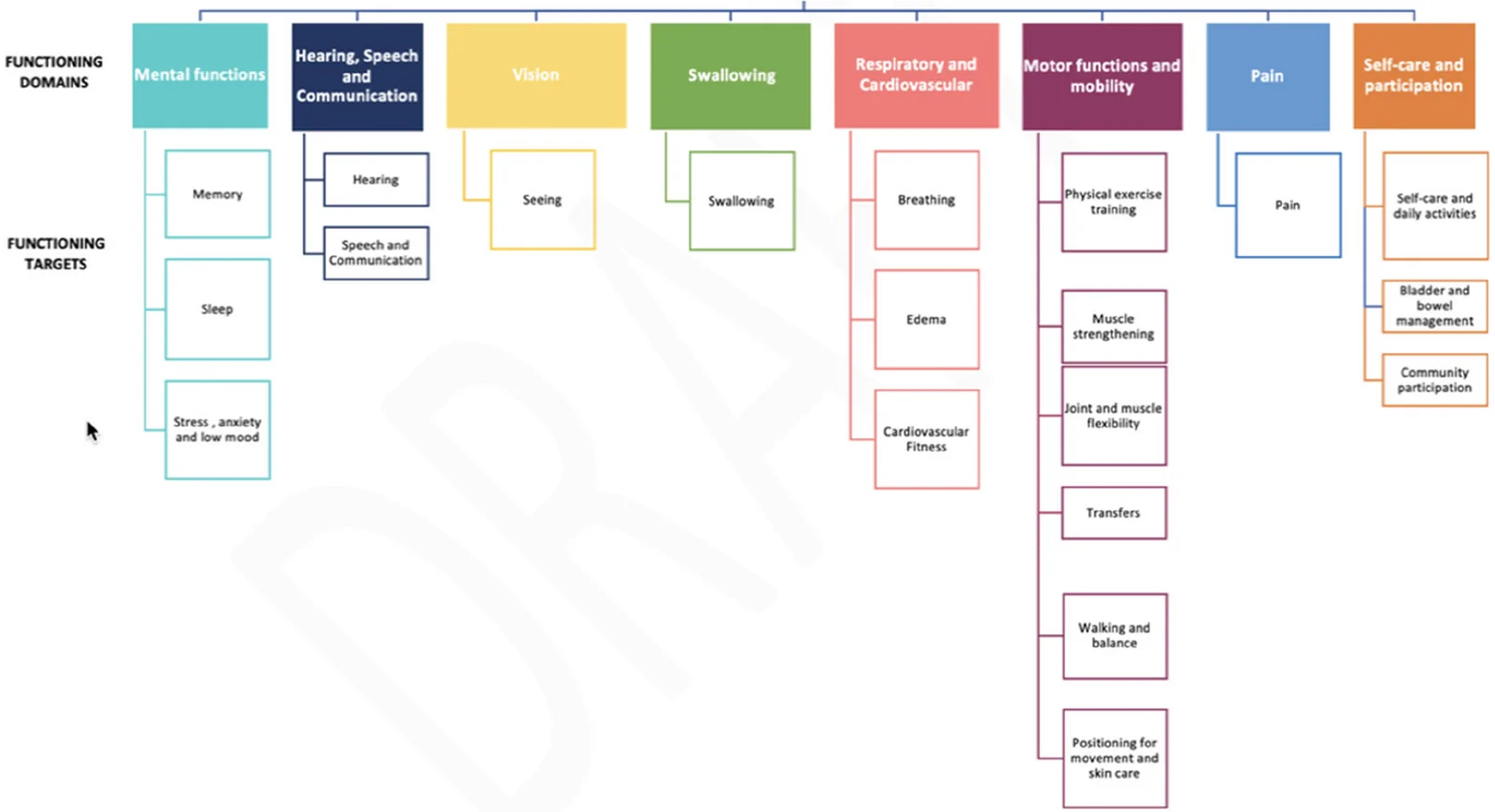
Overview of the eight functioning domains and the corresponding functioning targets for the Basic Package of Interventions for Rehabilitation

The structure (Fig. 2) of the Basic PIR clinical resource is composed of functioning domains and functioning targets. Broad functioning domains are broken down into specific functioning targets, each with corresponding interventions. The structure of the clinical resource was modelled after two existing WHO resources promoting task sharing in PC settings as templates: the WHO MH-GAP [41] and the WHO ICOPE [40].

For each functioning target, three clinical components were systematically drafted by the lead author: (1) clinical assessments to evaluate patient functional needs; (2) evidence-based "red flags" acting as strict referral criteria and contraindications; and (3) exercise, education, and patient advice modules. To populate these components with validated clinical data, a mapping was conducted using the verified Clinical Practice Guidelines and Cochrane Systematic Reviews previously utilized in the development of the parent PIR [46]. These components allowed for the implementation of targeted interventions tailored to the specific needs of individuals within each functioning target. They were developed as follows:

#### Component 1: Identification of assessment recommendations

The internal review panel identified, mapped and reviewed relevant clinical assessments based on their alignment with assessments from the existing ICOPE and mhGAP to ensure health system compatibility.

#### Component 2: Definition of red flags

To ensure safety in PC settings, criteria for situations where interventions should be contraindicated were identified by the lead author, forming the basis for the "red flags", and independently cross-verified by the internal review panel to ensure comprehensive safeguarding of patient well-being.

#### Component 3: Exercise, education and advice guidance

**Fig. 3:**
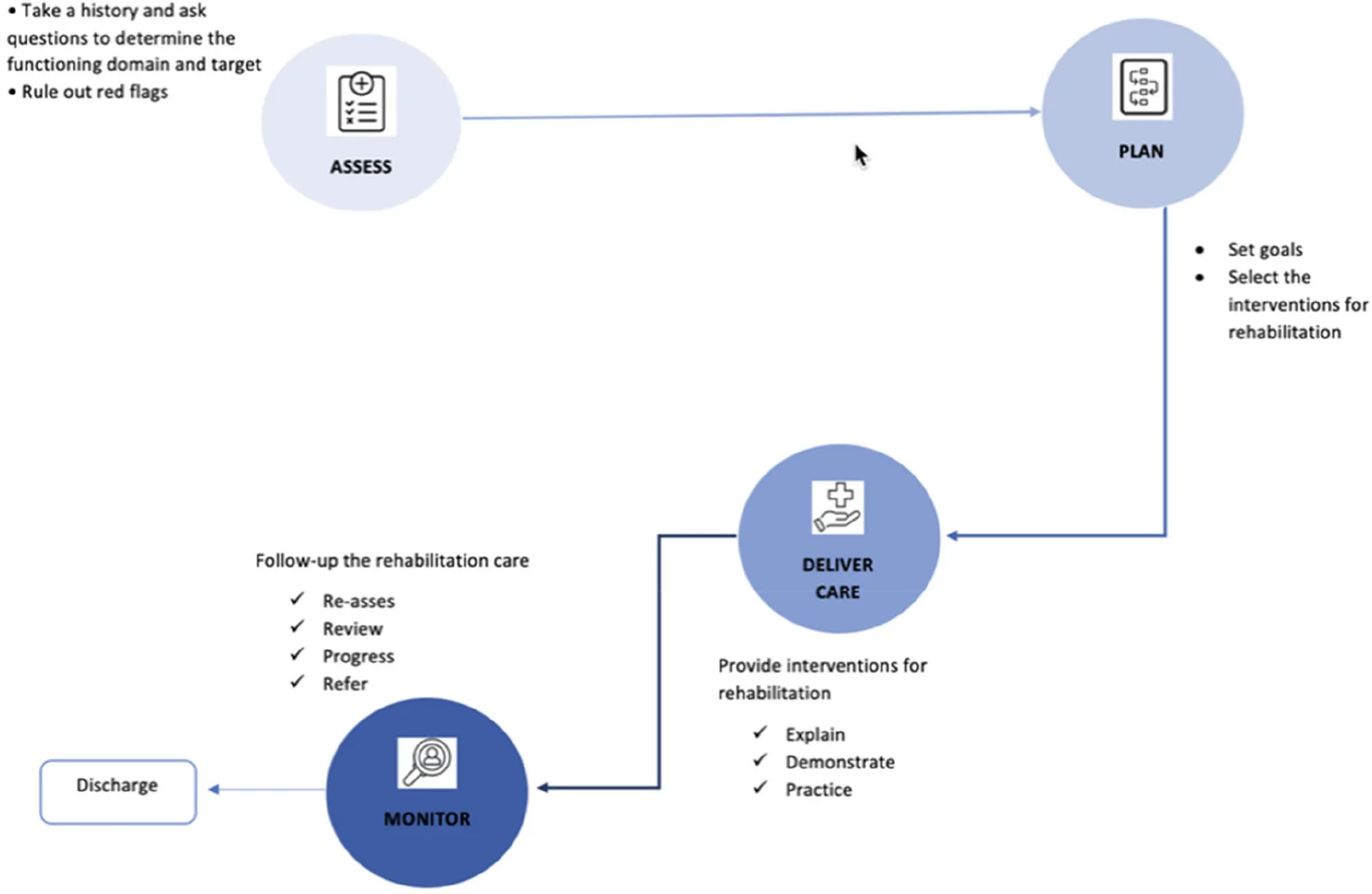
Four-step process for providing interventions for rehabilitation

The interventions for each functioning target were then grouped into two components: either exercise or education and advice for patients and their caregivers. This was to create a user-friendly, 4-step decision-tree model (Assess, Plan, Deliver Care, Monitor) (Fig. 3).

The five members of the internal review panel then independently evaluated the draft prototype via an online survey. The survey scores were compiled into a master matrix and followed by a web conference to consolidate comments. The draft was then distributed to the 26 TWG experts, who were divided into sub-panels based on their domain expertise. Text feedback on clarity and errors using a standardised from was collected from the experts. Finally, a joint web conference was held with all the TWG experts resolve any discrepancies and obtain a consensus on the prototype before field testing.

### Phase 3: Field test and review of the prototype

Two field tests were conducted to evaluate the prototype of the Basic PIR clinical resource.

#### First field tests and revision

The first field tests focused on two out of the eight functioning domains: motor functions and mobility; and self-care and participation. These studies were carried out in Ghana and Fiji in 2021 and 2022, respectively. The field tests comprised (1) in-country preparation, (2) in-country field test implementation, and (3) evaluation. The in-country preparation (1) involved stakeholder meetings to plan the field tests, whereas the implementation (2) entailed training PC non-rehabilitation workers through face-to-face lectures, practical sessions, and role-playing over a period of 5 days. The evaluation involved gathering feedback from the participants. After the 5-day training, a group discussion was held with the participants, during which their confidence in providing rehabilitation interventions on the basis of their training and the support needed in PC settings to enable them to provide rehabilitation was assessed. The participants’ feedback regarding the Basic PIR clinical resource structure and content was obtained by asking three questions (see supplementary file 1).

Verbal informed consent was obtained from participants in the preliminary field tests, and notes were taken during the discussions. This feedback was immediately analysed to identify key themes that informed the initial revision of the Basic PIR clinical resource. The revised resource then underwent a two-step review process. First, the internal review panel provided feedback via an online survey, followed by a web conference to consolidate comments. Then, the TWG reviewed the resource in groups based on their domain expertise, providing feedback on clarity, errors, and omissions. A final web conference was held with the TWG to discuss and approve the final revisions before pilot testing.

## Results

### Phase 1: Identification of priority interventions for rehabilitation

The initial phase of the study relied on two distinct expert bodies; the five-member internal review panel from the WHO Rehabilitation Programme and the 26-member multidisciplinary external TWG, the combined demographic profiles of which are detailed in Table 2.

**Table 2:** Demographics and expertise of study participants

**Participant ID**	**Gender**	**Job Title**	**Geographical Region**	**Self-Reported Expertise**	**Roles and responsibilities in product development**
**WHO Rehabilitation Programme Internal Review Panel**					
1	F	Consultant	AFRO	Rehabilitation/Primary care	Drafting, internal advisory and review
2	F	Technical Advisor	WPRO	Rehabilitation/Primary care/Health financing	Drafting, internal advisory and review
3	F	Consultant	EURO	Rehabilitation/Primary care/Workforce	Drafting, internal advisory and review
4	M	Consultant	EURO	Rehabilitation/Service Provision and standards	Internal advisory and review
5	F	Technical Advisor	EURO	Rehabilitation/Health Information systems	Internal advisory and review
**Technical Working Group (with panel assignments)**					
1	M	Audiologist	WPRO	Primary Care (Hearing, Speech and Communication)	TWG
2	F	General Practitioner/Community and Primary Care Physician	AFRO	Primary Care (Self-care and participation)	TWG
3	M	General Practitioner/Family physician	AFRO	Primary Care (Respiratory and Cardiovascular)	TWG
4	M	General Practitioner	EURO	Primary Care (Pain)	TWG
5	F	Nurse	AFRO	Primary Care (Self-care and participation)	TWG
6	F	Nurse	AFRO	Primary Care (Hearing, Speech and Communication)	TWG
7	M	Occupational therapist	AFRO	Rehabilitation (Mental functions)	TWG
8	M	Occupational therapist	AFRO	Rehabilitation (Motor functions and Mobility)	TWG
9	M	Occupational therapist	AFRO	Rehabilitation (Self-care and participation)	TWG
10	F	Ophthalmologist	WPRO	Primary Care (Seeing)	TWG
11	F	Ophthalmologist	EMRO	Primary Care (Seeing)	TWG
12	F	Optometrist	WPRO	Primary Care (Seeing)	TWG
13	M	Orthotist/Prosthetist	SEARO	Rehabilitation (Motor functions and mobility)	TWG
14	M	Orthotist/Prosthetist	SEARO	Rehabilitation (Pain)	TWG
15	F	Orthotist/Prosthetist	SEARO	Rehabilitation (Motor functions and mobility)	TWG
16	F	Physical and rehabilitation physician	WPRO	Rehabilitation (Swallowing)	TWG
17	M	Physical and rehabilitation physician	EMRO	Rehabilitation (Respiratory and Cardiovascular)	TWG
18	F	Physical and rehabilitation physician	PAHO	Rehabilitation (Respiratory and Cardiovascular)	TWG
19	F	Physical and rehabilitation physician	PAHO	Rehabilitation (Mental functions)	TWG
20	M	Physical therapist	SEARO	Rehabilitation (Motor functions and Mobility)	TWG
21	F	Physical therapist	SEARO	Rehabilitation (Pain)	TWG
22	F	Physical therapist	WPRO	Rehabilitation (Swallowing)	TWG
23	F	Physical therapist	AFRO	Rehabilitation (Pain)	TWG
24	M	Psychiatrist	AFRO	Rehabilitation (Mental functions)	TWG
25	F	Speech and language therapist/General Practitioner	WPRO	Rehabilitation (Swallowing)	TWG
26	F	Speech and language therapist	AFRO	Rehabilitation (Hearing, Speech and Communication)	TWG
**Field Test Participants - Ghana**					
1	M	Community Health Nurse	AFRO	Primary Care	Field Test Participant
2	F	Community Health Nurse	AFRO	Primary Care	Field Test Participant
3	M	Community Health Nurse	AFRO	Primary Care	Field Test Participant
4	F	Community Health Nurse	AFRO	Primary Care	Field Test Participant
5	M	Community Health Nurse	AFRO	Primary Care	Field Test Participant
6	F	Community Health Nurse	AFRO	Primary Care	Field Test Participant
7	M	General Nurse	AFRO	Primary Care	Field Test Participant
8	M	General Nurse	AFRO	Primary Care	Field Test Participant
9	M	General Nurse	AFRO	Primary Care	Field Test Participant
10	M	General Nurse	AFRO	Primary Care	Field Test Participant
11	F	General Nurse	AFRO	Primary Care	Field Test Participant
12	M	General Nurse	AFRO	Primary Care	Field Test Participant
13	F	General Nurse	AFRO	Primary Care	Field Test Participant
14	M	Mental Health Nurse	AFRO	Primary Care	Field Test Participant
15	M	Physician Assistant	AFRO	Primary Care	Field Test Participant
16	M	Physician Assistant	AFRO	Primary Care	Field Test Participant
17	F	Physician Assistant	AFRO	Primary Care	Field Test Participant
18	M	Physician Assistant	AFRO	Primary Care	Field Test Participant
19	M	Rehabilitation Assistant	AFRO	Primary Care	Field Test Participant
**Field Test Participants - Fiji**					
1	F	Community Rehabilitation Assistant	WPRO	Primary Care	Field Test Participant
2	F	Community Rehabilitation Assistant	WPRO	Primary Care	Field Test Participant
3	M	Medical Officer	WPRO	Primary Care	Field Test Participant
4	F	Nurse	WPRO	Primary Care	Field Test Participant
5	F	Nurse	WPRO	Primary Care	Field Test Participant
6	F	Nurse Practitioner	WPRO	Primary Care	Field Test Participant
7	F	Nurse Practitioner	WPRO	Primary Care	Field Test Participant
8	F	Nurse Practitioner	WPRO	Primary Care	Field Test Participant
9	F	Physiotherapist	WPRO	Primary Care	Field Test Participant
10	M	Physiotherapist	WPRO	Primary Care	Field Test Participant
11	F	Stroke Nurse	WPRO	Primary Care	Field Test Participant
12	F	Zone Nurse	WPRO	Primary Care	Field Test Participant
13	F	Zone Nurse	WPRO	Primary Care	Field Test Participant
14	F	Zone Nurse	WPRO	Primary Care	Field Test Participant
15	F	Representative – Fiji Cancer Society	WPRO	Primary Care	Field Test Participant
16	M	Representative – Diabetes Fiji Inc	WPRO	Primary Care	Field Test Participant
17	F	Representative – Wellness Fiji	WPRO	Primary Care	Field Test Participant

*AFRO* WHO Regional Office for Africa, *AMRO/PAHO* WHO Regional Office for the Americas/Pan American Health Organization, *EMRO* WHO Regional Office for the Eastern Mediterranean, *EURO* WHO Regional Office for Europe, *SEARO* WHO Regional Office for South-East Asia, *WPRO* WHO Regional Office for the Western Pacific

The health conditions selected to establish the framework of the Basic PIR are shown in Table 3 (boldened rows).

**Table 3:** Health conditions selected for the Basic Package of Interventions for Rehabilitation (Basic PIR) on the basis of global prevalence, disability weights and years lived with disability) data. The selected health conditions are boldened

**Disease area**	**Health conditions included in the PIR**^**1**^	**Global prevalence (in thousands)**	**Disability weights**^**2**^	**Years lived with Disability (in thousands)**	**Health conditions included in the Basic PIR (Y/N)**
Mental health disorders	**(Major) Depressive disorders**	**163044**	**0.145–0.658**	**32846**	**Y**
	Schizophrenia	19776	0.588–0.778	12657	N
	Idiopathic developmental intellectual disability	100572	0.043–0.2	4046	N
	Autistic spectrum	31179	n.a.	4731	N
Musculoskeletal conditions	**Low back pain**	**576980**	**0.02–0.372**	**64946**	**Y**
	**Osteoarthritis**	**303096**	**0.023–0.165**	**9604**	**Y**
	Sarcopenia	*n.a.*	*n.a.*	*n.a.*	N
	Rheumatoid arthritis	19965	0.117–0.581	2626	N
	**Fracture**	**360136**	**n.a.**	**19793**	**Y**
	Amputation	371422	n.a.	640	N
Neurological conditions	**Cerebrovascular disease (Stroke)**	**104178**	**0.019–0.588**	**18695**	**Y**
	Traumatic brain injury	n.a.	n.a.	n.a.	N
	Spinal cord injury (Global Burden of Disease 'Spinal injuries')	22489	0.296 (Paraplegic)	6633	N
	Alzheimer’s disease and other dementias	44988	0.069–0.449	6570	N
	Parkinson’s disease	8525	0.01–0.575	1219	N
Cardiovascular conditions	**Ischemic heart disease**	**126451**	**0.033–0.179**	**5291.0**	**Y**
Respiratory conditions	**Chronic obstructive pulmonary disease**	**299398**	**0.019–0.408**	**30611**	**Y**
Child health	**Neonatal encephalopathy due to birth asphyxia and trauma including Cerebral Palsy**	**53823**	**n.a.**	**9703**	**Y**
Sensory conditions	**Hearing impairment**	**1428450**	**0.01–0.215**	**39454**	**Y**
	**Vision impairment**	**1339970**	**0.003–0.187**	**34125**	**Y**
Neoplasm	Breast cancer (Cancer)	16697	Diagnosis & primary therapy phase: 0.288 Controlled phase: 0.049 metastatic phase: 0.451 Terminal phase: 0.54	1307	N
**Disease area**	Health conditions included in the PIR^a^	Global prevalence (in thousands)	Disability weights^a^	Years lived with Disability (in thousands)	Health conditions included in the Basic PIR (Y/N)
Mental health disorders	**(Major) Depressive disorders**	**163044**	**0.145–0.658**	**32846**	**Y**
	Schizophrenia	19776	0.588–0.778	12657	N
	Idiopathic developmental intellectual disability	100572	0.043–0.2	4046	N
	Autistic spectrum	31179	n.a.	4731	N
Musculoskeletal conditions	**Low back pain**	**576980**	**0.02–0.372**	**64946**	**Y**
	**Osteoarthritis**	**303096**	**0.023–0.165**	**9604**	**Y**
	Sarcopenia^a^	*n.a.*	*n.a.*	*n.a.*	N
	Rheumatoid arthritis	19965	0.117–0.581	2626	N
	**Fracture**	**360136**	**n.a.**	**19793**	**Y**
	Amputation	371422	n.a.	640	N
Neurological conditions	**Cerebrovascular disease (Stroke)**	**104178**	**0.019–0.588**	**18695**	**Y**
	Traumatic brain injury	n.a.	n.a.	n.a.	N
	Spinal cord injury (Global Burden of Disease 'Spinal injuries')	22489	0.296 (Paraplegic)	6633	N

^1^PIR: Package of Interventions for Rehabilitation

^2^Disability weights sourced from IHME 2017 data resources: Global Burden of Disease 2017 Disability weights. Accessed here: http://ghdx.healthdata.org/gbd-2017

^3^No data was available for this health condition in Global Burden of Disease 2016 or 2017. This health condition were selected by rehabilitation experts during the PIR development process, as they were deemed highly relevant for rehabilitation in low- and middle-income countries

Table 3: Health conditions selected for the Basic Package of Interventions for Rehabilitation (Basic PIR) on the basis of global prevalence, disability weights and years lived with disability (YLD) data. The selected health conditions are boldened.

To ensure the Basic PIR targets functioning, a total of 13 functioning domains linked to 10 out of the 20 health conditions were defined (Table 4).

**Table 4:** Comparison of the selected health conditions against the 13 functioning domains selected from the Package of Interventions for Rehabilitation (PIR) for the Basic PIR

Functioning Domain	Stroke	CP	LBP	OA	Fractures	COPD	IHD	Depression	Vision	Hearing
Mental and Cognitive functions	**✓**	**✓**	**✓**			**✓**	**✓**	**✓**		**✓**
Vision	**✓**	**✓**							**✓**	
Speech, Language and Communication	**✓**	**✓**								**✓**
Hearing and Vestibular functions		**✓**								**✓**
Dysphagia management	**✓**	**✓**								
Nutrition	**✓**	**✓**				**✓**		**✓**		
Pain management	**✓**	**✓**	**✓**	**✓**	**✓**					
Bowel and bladder management	**✓**	**✓**								
Respiration functions		**✓**			**✓**	**✓**	**✓**			
Cardiovascular and immunological functions	**✓**	**✓**			**✓**		**✓**			
Motor functions and Mobility	**✓**	**✓**	**✓**	**✓**	**✓**	**✓**	**✓**	**✓**		
Activities of Daily Living	**✓**	**✓**	**✓**	**✓**	**✓**	**✓**		**✓**	**✓**	**✓**
Community and social life	**✓**	**✓**	**✓**	**✓**		**✓**	**✓**	**✓**	**✓**	**✓**

*CP* Cerebral palsy, *LBP* Low back pain, *OA* Osteoarthritis, *COPD* Chronic obstructive pulmonary disease, *IHD* Ischemic heart disease

**Fig. 4:**
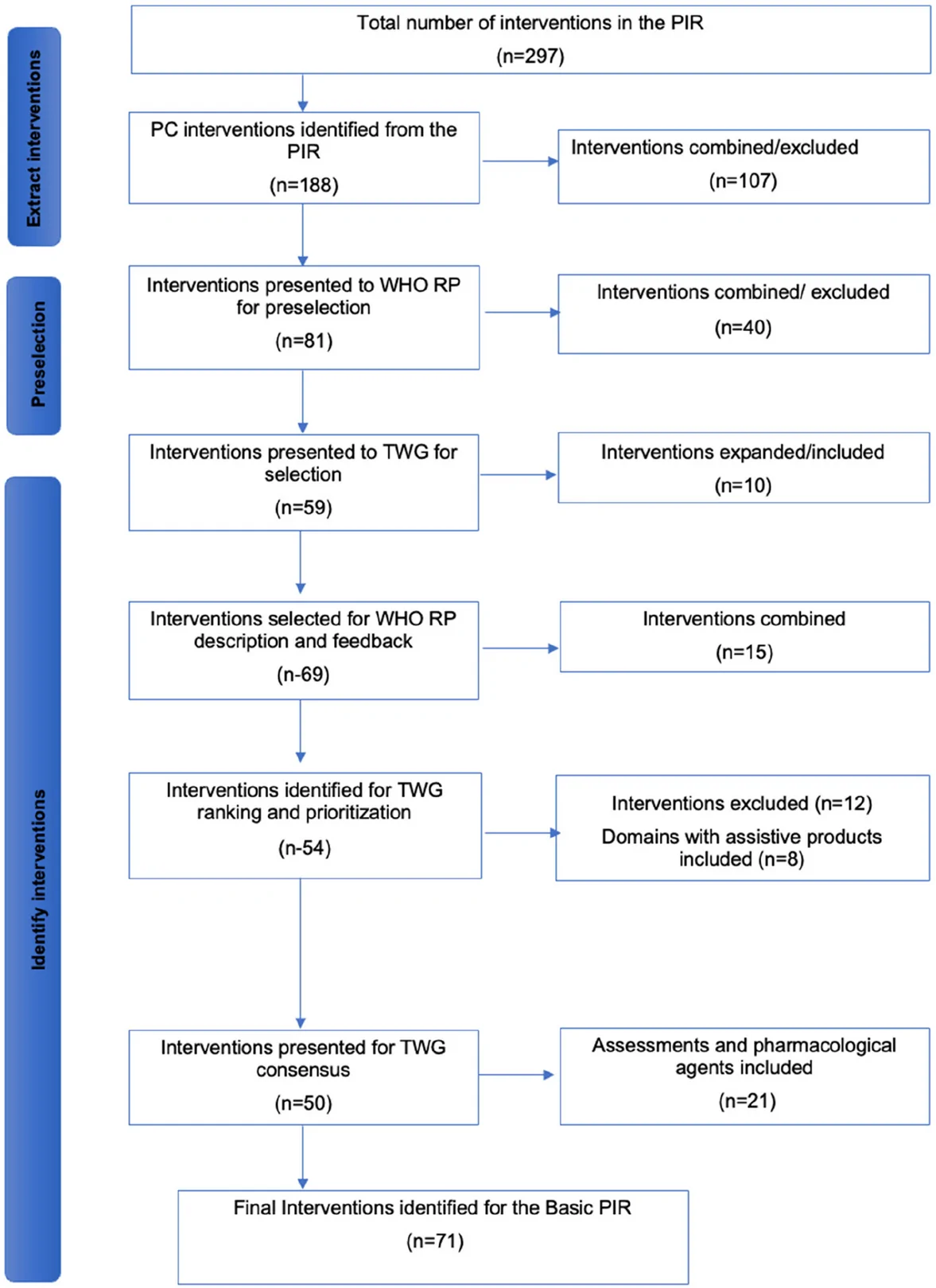
Flow chart of the selection of interventions for the Basic PIR

As shown in the flowchart (Fig. 4), the Basic PIR's interventions were selected through a structured process. Starting with 297 interventions from the PIR, 188 were pre-selected for primary care suitability. From this group, 107 were either combined or excluded due to redundancy or infeasibility in a PC setting (e.g., comprehensive diet management was excluded). After revision by the internal review panel, 59 interventions were presented to the TWG, who recommended adding 10 more, such as advice on safe swallowing for dysphagia. After further ranking and prioritization, and the inclusion of 11 assistive product interventions and 21 assessments and pharmacological agents, the final list for the Basic PIR consisted of 71 interventions.

### Phase 2: Drafting the prototype

The PIR functioning domains, the Basic PIR functioning domains, the functioning targets and the corresponding interventions are described in Table 5.

**Table 5:** Package of Interventions for Rehabilitation (PIR) functioning domains, the Basic PIR functioning domains, functioning targets and their corresponding ICF, ICD-11 codes for functioning properties and interventions

**PIR Functioning domains**	**Basic PIR Functioning domains**	**Basic PIR Functioning targets**	**ICF**	**ICD-11**	**Basic PIR Interventions**
1. Mental and Cognitive functions	1. Mental functions	1. Memory	b144 Memory functions	VV03 Memory functions	1. Assessment of memory functions 2. Basic memory training 3. Provision and training in the use of assistive products for memory functions (simple memory supports, pill organizers)
		2. Sleep	b134 Sleep functions	VV01 Sleep functions	4. Assessment of sleep functions 5. Education and advice on self-management of sleep disturbances
		3. Stress, anxiety and low mood	b152 Emotional functions d240 Handling stress and other psychological demands	VV04 Emotional functions	6. Assessment of stress, anxiety and low mood 7. Providing psychological support 8. Education and advice on self-management of anxiety and low mood 9. Education and advice on self-management of stress
2. Vision	2. Seeing	4. Seeing functions	b210-b229 Seeing and related functions	VV10 Seeing and related functions	10. Assessment of seeing functions 11. Education and advice on modification of the home environment 12. Education and advice on self-management of vision impairment 13. Provision and training in the use of assistive products for vision (eg. spectacles, magnifiers and telescopes) 14. Provision and training in the use of assistive products for reading and writing (eg. braille books, communication boards) 15. Provision and training in the use of assistive products for mobility (eg. white canes)
3. Hearing and Vestibular functions 4. Speech, language and Communication	3. Hearing, Speech, and Communication	5. Hearing	b230-b249 Hearing and vestibular functions	VV11 Hearing and vestibular functions	16. Assessment of hearing functions 17. Education and advice on self-management of hearing impairment 18. Provision and training in the use of assistive products for hearing (alarm signalers, hearing aids, personal microphone systems)
		6. Speech and Communication	b3 Voice and speech functions	VV2Y Other specified voice and speech functions VV2Z Voice and speech functions unspecified	19. Assessment of speech functions, language and communication 20. Basic speech therapy 21. Basic language therapy 22. Basic communication skills training 23. Education and advice on self-management of speech, language and communication difficulties 24. Provision and training in the use of assistive products for communication (communication boards, books and software, simplified mobile phones)
5. Nutrition 6. Dysphagia management	4. Swallowing	7. Swallowing	b5105 Swallowing	VV40 Functions related to the digestive system	25. Assessment of swallowing 26. Basic swallowing therapy 27. Education and advice on self-management of swallowing difficulties 28. Provision of pharmacological agents for swallowing management (Proton pump inhibitor, botulinum toxin A) 29. Basic nutritional management training
7. Respiration functions 8. Cardiovascular and Immunological functions	5. Respiratory and Cardiovascular	8. Breathing	b440 Respiration functions	VV3Y Other specified functions of the cardiovascular, haematological, immunological and respiratory systems	30. Assessment of breathing functions 31. Training in basic breathing control techniques 32. Education and advice on self-management of breathlessness 33. Training in basic energy conservation techniques
		9. Edema	b435 Immunological system functions	VV3Y Other specified functions of the cardiovascular, haematological, immunological and respiratory systems	34. Assessment of edema 35. Training in basic retrograde massage techniques 36. Training in positioning for oedema control 37. Basic range of motion exercises for oedema control 38. Provision and training in the use of assistive products for compression therapy
		10. Cardiovascular fitness	b410 Heart functions b455 Exercise tolerance functions	VV30 Exercise tolerance functions	39. Assessment of heart and blood pressure functions 40. Education and advice on self-management of cardiovascular diseases
9. Motor functions and Mobility	6. Motor functions and Mobility	11. Physical exercise training	b455 Exercise tolerance functions	VV30 Exercise tolerance functions	41. Assessment of exercise capacity 42. Basic physical exercise training
		12.Muscle strengthening	b730 Muscle power functions	VV61 Muscle power functions VW12 Carrying, moving and handling objects	43. Assessment of muscle functions 44. Basic muscle strengthening training
		13. Joint and muscle flexibility	b710 Mobility of joint functions	VV60 Mobility of joint functions VW12 Carrying, moving and handling objects	45. Assessment of muscle and joint flexibility 46. Basic range of motion exercises
		14. Gait (Walking) and Balance	d450-d469 Walking and moving	VW13 Walking VW14 Moving around within the home VW15 Moving around using equipment	47. Assessment of walking and balance 48. Basic gait (walking) and balance training 49. Provision and training in the use of assistive products for mobility- walking and balance (eg. crutches, walkers)
		15.Positioning for movement and skin care	d410-d429 Changing and maintaining body position	VW10 Maintaining a standing position	50. Assessment of positioning 51.Training in positioning for the prevention of contracture 52. Training in positioning for pressure relief 53. Provision and training in the use of assistive products for pressure relief (eg. pressure relief cushions)
		16. Mobility (Transfers)	d420 Transferring oneself	VW11 Transferring oneself	54. Assessment of mobility (transfers) 55. Basic mobility (transfers) training 56. Provision and training in the use of assistive products for mobility-transfers (eg. transfer boards)
10. Pain management	7. Pain	17.Pain	b280-b289 Pain	VV12 Sensation of pain	57. Assessment of pain 58. Basic thermotherapy 59. Basic range of motion exercises for pain management 60. Education and advice on self-management of pain 61. Provision of pharmacological agents for pain management (eg. Analgesics)
11. Activities of Daily Living 12. Bowel and bladder management 13. Community and Social life	8. Self-care and Participation	18. Self-care and daily activities	d5 Self-care d630-d649 Household tasks	VW20 Washing oneself VW21 Caring for body parts VW22 Toileting VW23 Dressing VW24 Eating VW16 Using transportation VW30 Preparing meals VW31 Doing housework VW32 Assisting others	62. Assessment of activities of daily living and self-care 63. Education and advice on self-care and management of activities of daily living 64. Provision and training in the use of assistive products for self-care (eg. Toilet and shower chairs)
		19. Bladder and bowel management	b620 Urination functions	VV50 Urination functions VV40 Functions related to the digestive system	65. Assessment of urination and defecation functions 66. Education and advice on self-management of bowel and bladder functions 67. Basic pelvic floor exercises 68. Provision of pharmacological agents for bladder and bowel management (eg. Laxatives) 69. Provision and training in the use of incontinence products
		20. Community and social participation	d910 Community life d920 Recreation and leisure	VW60 Recreation and leisure	70. Assessment of participation in community and social life 71. Education and advice on self-management of participation in community and social life

Table 5: Package of Interventions for Rehabilitation (PIR) functioning domains, the Basic PIR functioning domains, functioning targets and their corresponding ICF, ICD-11 codes for functioning properties and interventions.

### Phase 3: Field test and review of the prototype

The field test involved 19 health workers in Ghana and 17 in Fiji from various PC settings nationwide (Table 2).

Appendix B summarizes all the feedback received and the corresponding modifications. For example, feedback from primary care workers in Ghana noted the difficulty in distinguishing between the 'Plan' and 'Deliver Care' steps of the 4-step process. In response, the process was simplified to a 3-step model where the 'Plan' stage was merged into the 'Deliver care' phase. Similarly, health workers in Fiji highlighted the need to grade functioning difficulty to better track progress. This led to the development of a 0–10 grading system for each functioning domain, as well as patient rehabilitation handouts with illustrations to guide the provision of interventions.

## Discussion

This article aimed to describe the participatory development process of the list of interventions and clinical resource of the Basic PIR, a tool for integrating rehabilitation in PC settings through task sharing. The Basic PIR was developed on the basis of the WHO's PIR [46], a comprehensive list of interventions designed to support countries in integrating rehabilitation into their health systems. Field tests of the prototype conducted in Ghana and Fiji provided preliminary insights into the content of the Basic PIR clinical resource and informed its revision, including the simplification of the assessment process and the development of user-friendly tools, such as patient handouts. These modifications are intended to enhance the usability and clarity of the Basic PIR for PC non-rehabilitation workers, though their direct impact on clinical patient outcomes remains to be empirically evaluated. The Basic PIR aims to empower existing PC non-rehabilitation workers, such as doctors and nurses, to deliver basic rehabilitation interventions via a carefully selected set of prioritised and evidence-based interventions chosen for their safety, effectiveness, and ease of learning. The acceptability, appropriateness and feasibility of the Basic PIR will be evaluated in a series of pilot studies across multiple countries.

As a toolkit, the Basic PIR is intended to contribute to the goals of the World Health Assembly resolution by improving the baseline availability and accessibility of basic rehabilitation services, particularly in primary care [47]. The Basic PIR has been designed to complement, rather than substitute existing PC tools and whole-sector rehabilitation strengthening approaches. One of the key features of the Basic PIR is the identification and prioritization of rehabilitation interventions that can be safely and effectively delivered by a non-rehabilitation workforce in PC settings through task sharing. A similar approach has been used in the development of two WHO-established resources: ICOPE [42] and mhGAP [43]. The WHO's mhGAP, for example, employs a task-sharing approach to address the substantial gap between the need for and availability of mental, neurological, and substance use services. A systematic review, however, identified several challenges in implementing mhGAP, including a shortage of specialist staff for supervision, negative cultural attitudes toward mental health, and competing demands on primary care workers’ time [48]. Similarly, the ICOPE initiative provides guidance for delivering person-centred care to older adults in primary care settings. While ICOPE has demonstrated significant benefits, it has encountered challenges such as mhGAP, including workforce capacity issues [48, 49]. Additionally, PC providers have reported difficulties conducting the necessary assessments and interventions after initial screening [48, 49]. These experiences were considered during the development of the Basic PIR. They highlight the importance of simplified interventions that are easy for non-rehabilitation PC workers to identify and have a feasible time to deliver in PC settings. However, we acknowledge the risk that the deployment of the Basic PIR will face similar systemic bottlenecks regarding workforce shortages and supervisory capacity. The planned pilot testing will therefore evaluate both the feasibility of delivering these interventions within existing workloads and the acceptability of the approach to PC workers, providing data to further refine strategies for managing workforce capacity in real-world settings.

The Basic PIR underwent a multi-stage development process aimed at maximizing multidisciplinary input while identifying potential structural biases. The participatory approach applied via a modified Delphi process with input from a diverse group of rehabilitation and non-rehabilitation healthcare experts ensured that the selected interventions were not only evidence-based but also feasible for implementation in PC and resource-limited settings. The emphasis on cost-effective, high-impact interventions that are easy to learn and integrate into existing workflows aligns with global recommendations and guidelines for task sharing [50]. This approach aims to integrate basic rehabilitation into existing workflows rather than adding a new, separate task. Ultimately, the aim is to lower the barrier to deliver basic rehabilitation services at the PC level, though it represents only an introductory step toward addressing the broader workforce capacity challenges encountered by previous WHO initiatives such as the WHO’s mhGAP and ICOPE.

Person-centred care is crucial in primary care [51]. While the Basic PIR uses a structured approach, its focus on functioning, defined as the human experience related to body functions, structures, activities, and participation [52] is intended to support person-centred care. The functioning domains selected for the Basic PIR correspond to the ICF codes integrated into the International Classification of Diseases 11^th^ Revision (ICD-11) (see Table 5). While this alignment supports basic standardization, it is important to note that the tool captures only a narrow subset of PC functioning domains.

By considering a person's abilities and needs within their environment, the tool allows health workers to provide a more tailored consultation and empower patients with self-management education. Indeed, a recent stakeholder survey revealed that while inpatient and outpatient rehabilitation models were most frequently reported as disease-centred, rehabilitation provided at home, in the community, and in eldercare was more functioning-centred [53]. The Basic PIR is designed to complement these community-oriented contexts. Health care workers in most low-resource, rural or remote PC settings also support a functioning-based approach, recognizing that in these settings, individuals with functioning needs may not have a formal diagnosis owing to limited access to trained health professionals [54, 55]. A brief functioning assessment, which is relatively straightforward can help guide the provision of interventions for rehabilitation [54]. This approach aligns with current healthcare trends that prioritize the capacity of a person to perform daily activities as a central aspect of health and well-being [56]. In this sense, the Basic PIR concentrates on enhancing functioning within the PC context. Furthermore, this functioning-focused approach lays the groundwork for seamless integration with primary care classification systems, such as the International Classification of Primary Care. Moving from a purely condition-focused system to one that captures functioning is essential for defining service needs and improving data quality in primary care[57]. Healthcare workers accustomed to a condition-focused approach to rehabilitation, especially those working in low-resource settings without access to formal diagnosis, may however need to adopt a new mindset when using the function-focused Basic PIR. Content from the Basic PIR are however restricted to low-complexity domains and should not be interpreted as reflecting broader rehabilitation service requirements.

The Basic PIR may also serve as a resource for rehabilitation specialists particularly in understanding the scope of interventions utilized in PC task-sharing. This mutual understanding can enable specialists to provide targeted supervision, technical mentorship, and streamlined integration into broader health systems. Integrating PC interventions into professional development can also be beneficial for rehabilitation specialists at all levels of healthcare [58]. By emphasizing functioning and the impact of functioning difficulties on daily life, the Basic PIR is designed to enhance the focus on the patient's perspective, functional outcomes, and collaboration with patients and caregivers. This tool is designed to support a shift away from a purely disease-centric medical model to a functioning-based, person-centred approach regardless of the care setting. It also complements efforts to address an individual's needs within their environment. While the tool primarily focuses on body functions, body structures, activities and participation, it also serves as a clinical resource by including interventions and advice on environmental modifications. For instance, the vision section provides education and advice on modification of the home environment. However, a potential risk of the Basic PIR is that individuals with conditions requiring specialized care may remain in PC. To mitigate this risk, the Basic PIR clinical resource includes clear referral pathways and a "red flags" section to guide PC non-rehabilitation workers in identifying when and how to refer patients to specialists. Additionally, involving rehabilitation specialists as mentors remains a necessity to prevent clinical stagnation and ensure that complex cases are safely escalated through established referral networks.

At the system level, the Basic PIR should be viewed strictly as a preliminary resource for initial PC task-sharing, not a blueprint for workforce planning or resource allocation. Because it only addresses low-complexity interventions, using it in isolation risks severely underestimating national infrastructure and specialized staffing needs. Similarly, while the toolkit identifies low-cost options for underserved areas, it must not define or limit national essential health benefit packages. Doing so risks narrowing the scope of services to a lean, incomplete set of functional targets while neglecting complex acute, secondary, and tertiary care needs. Furthermore, any data monitoring supported by this tool is inherently restricted to its specific domains and cannot constitute or substitute a robust, nationwide health information system. Therefore, to safeguard the full continuum of care, the Basic PIR must be applied transparently, acknowledging its developmental biases. It should function only as a complementary baseline component nested within a broader health system that actively integrates specialized rehabilitation evidence, comprehensive standard treatment guidelines, and robust referral networks.

The development of the Basic PIR has several limitations. The first is the potential for developer bias. The five internal review panel members were central to both the preliminary review and the tool's development, including the selection of Delphi participants, which may have influenced the perspectives represented. Additionally, the panel leant geographically toward the EURO region. To address this, an external TWG was recruited, ensuring geographic equity across all six WHO regions and balancing specialist perspectives with perspectives from frontline healthcare workers in PC and low-resource settings. Furthermore, the evidence base relied heavily on existing WHO tools and documents rather than a comprehensive literature review, potentially limiting the breadth of sources considered.

A second limitation is that the field tests were constrained. They concentrated on only two of the eight functioning domains and involved a small number of health workers from just two countries. Because the feedback was collected immediately after training via group discussions (with no application of strategies in a clinical setting), client outcomes and satisfaction were not assessed. This limits the generalizability of our findings and the insights into the tool’s real-world effectiveness.

Furthermore, we acknowledge the significant challenge of resource availability in low-income settings. The Basic PIR is designed to be a flexible tool that optimizes the use of existing resources. While it includes a list of interventions that may require specific resources, it prioritizes those that are low-cost, readily available, and can be implemented with minimal equipment. The assistive products mentioned are from the WHO Priority Assistive Products List, which identifies essential, affordable, and quality-assured products. The tool's aim is not to introduce a new and expensive set of demands but to provide guidance on the most effective interventions within the realistic scope of primary care workers.

Finally, a formal scoping review was not conducted, meaning the initial selection of health conditions may have limited the study’s scope and potentially overlooked some relevant functioning domains. We note, however, that the tool's focus on functioning allows for its application to a broader range of health conditions beyond the initially selected ones.

## Conclusion

The Basic PIR might be a promising step toward improving access to rehabilitation services in PC and low-resource settings. By leveraging task-sharing and focusing on prioritised, high-impact, and cost-effective interventions, this context-sensitive toolkit has the potential to help bridge the gap in rehabilitation services and contribute to the achievement of UHC.

The development process demonstrates the value of stakeholder engagement; continuous feedback from local health workers directly informed the simplification of the intervention delivery steps, the creation of a patient functional grading system, and the design of illustrated patient handouts to maximize real-world usability.

Future pilot studies will use implementation research to evaluate the Basic PIR’s operational feasibility, acceptability, and appropriateness in PC and low-resource settings. These studies will also gather feedback from PC workers on the task-sharing model and explore the potential for a collaborative approach with rehabilitation specialists to support long-term sustainability.

## Supplementary Information

Supplementary Material 1. Supplementary file 1: Discussion questions for primary care non-rehabilitation worker feedback. 1. Are there any features you find confusing or difficult to use? 2. Is anything missing? 3. Are the instructions practical and easy to implement?

## Data Availability

The datasets used and/or analyzed during the current study are available from the corresponding author on reasonable request.

## Appendix A

### Plain language summary

Introduction

- Approximately 2.6 billion people globally need rehabilitation services, yet access is severely limited, especially in rural and low-resource communities.
- The main obstacle is a critical shortage of specialized rehabilitation professionals in primary care (PC) settings.
- The World Health Organization (WHO) developed the Basic Package of Interventions for Rehabilitation (Basic PIR) to address this gap.

Methods

- The goal is to empower local healthcare workers (PC non-rehabilitation workers) to safely and effectively deliver basic rehabilitation services through task-sharing, rather than taking tasks away from specialized professionals, which helps address provider shortages in many settings.
- A diverse Technical Working Group (TWG) of 26 experts from six WHO regions was involved in the three-phase development process. The TWG reviewed 297 potential options and prioritised 71 essential interventions that are easy to learn, safe, and feasible to deliver during routine patient care using low-cost equipment.
- Preliminary field tests were conducted in Ghana and Fiji, leading to simplified assessment steps and the creation of clear, illustrated patient handouts.

Results

- The Basic PIR shifts care from a "disease-centred" model to a "functioning-centred" model, helping workers assess a person’s abilities and needs related to eight functioning domains (e.g., mobility, vision, mental functions).
- The resource includes clear"red flags" and referral criteria to guide PC workers on when a patient must be immediately referred to a specialist, ensuring patient safety.
- This focus on functioning aligns the tool with global data standards, specifically ICF codes integrated into the ICD-11

Discussion and conclusion

- The Basic PIR provides a practical, evidence-based toolkit to successfully integrate basic rehabilitation services into primary care where specialists are unavailable. This is a major step toward achieving Universal Health Coverage (UHC).
- Future studies will evaluate the tool’s real-world appropriateness, acceptability and feasibility as well as its ultimate impact on patient outcomes.

## Appendix B

### Group feedback and modifications of the Basic PIR

**Table:**

**Group**	**Feedback solicited**	**Feedback obtained**	**Modifications made**
Ghana Primary Care Workers	Structure and content of the Basic PIR clinical resource - Steps to providing rehabilitation (Assess, Plan, Deliver Care, Monitor) -Components of functioning targets (assessments, red flags, exercise, education, and advice interventions).	• Difficulty with making a distinction between ‘Plan’ and ‘Deliver care’ aspect of the 4-step process • Difficulty remembering all the questions to ask a client	• The 4-step process was simplified to a 3-step process with ‘Plan’ stage merged into the ’Deliver phase’. • An assessment form developed to easily guide the ‘Assess’ stage
Fiji Primary Care Workers		• It would help to be able to grade the level of functioning difficulty to compare with levels during subsequent visits • Having a handout to guide the provision of interventions could be a helpful tool for prompting memory and consistency.	• Grading of level of difficulty for each functioning domain was developed with a grading system of ‘0–10’ • Patient rehabilitation handouts with illustrations and simple instructions for each functioning domain developed. Patients could take a photo, photocopy or print it out to use at home.
WHO Rehabilitation Programme		• Including a form for easy identifications of clinical presentations that could benefit from rehabilitation will shorten the time required to provide rehabilitation interventions in PC • Red flags are an important component and should be stressed upon • In situations where there is a confirmation of the health condition, information on how to manage the health condition should be provided	• Consolidation of common clinical presentations for each rehabilitation domain into one form for easy reference. • Red flags for each rehabilitation domain consolidated on one form for easy reference and use • Patient rehabilitation handouts were developed for the 10 health conditions that were used to guide the initial selection of interventions from the PIR.
Technical Working Group	Content of the Basic PIR clinical resource -Components of functioning targets (assessments, red flags, exercise, education, and advice interventions). Questions guiding the provision of feedback - Are the instructions easy to understand? - Are there any errors? - Is there anything missing? - Are there any other comments?	• Red flags – add: -sudden onset hearing loss accompanied by vertigo for hearing -red eye for vision -choking and coughing for swallowing • Assessments – add: - Hearing difficulty that affects ability to converse in different environment to common clinical presentations for hearing • Interventions a. Add - Education on the impact of improved hearing being ability to communicate with family, coworkers, and friends (social impact of improved hearing) - Use of a sign or gestures can improve communication -Advice on reviewing the environmental modifications and suggesting any changes if needed to vision interventions b. Modify -‘Adopt a good posture’ in pain interventions to ‘Frequently changing positions can result in pain relief to pain’ - The sequence of swallowing interventions to begin with neck strengthening exercises and end with advice on eating the appropriate food texture	All suggested modifications were accepted during the web conference following which modifications were made to the Basic PIR clinical resource.

## Abbreviations

AFRO: WHO Regional Office for Africa
Basic PIR: Basic Package of Interventions for Rehabilitation
EMRO: WHO Regional Office for the Eastern Mediterranean
EURO: WHO Regional Office for Europe
ICF: International Classification of Functioning, Disability, and Health
ICOPE: Integrated Care for Older People
mhGAP: Mental Health Gap Action Programme for mental, neurological and substance use disorders in non-specialist health settings
NCDs: Noncommunicable diseases
PAHO: WHO Regional Office for the Americas
PC: Primary Care
PIR: Package of Interventions for Rehabilitation
SEARO: WHO Regional Office for South-East Asia
TWG: Technical Working Group
UHC: Universal Health Coverage
WHO: World Health Organization
WPRO: WHO Regional Office for the Western Pacific
