# Supplementary material for: Strengthening primary care with the world health organization’s basic package of interventions for rehabilitation: developing a list of interventions and a clinical resource
Source: BMC Prim Care. 2026 Oct 16;27:373. doi: 10.1186/s12875-026-03479-z (PMC13673863; doi:10.1186/s12875-026-03479-z)
Supplement: Supplementary file 1 [file 12875_2026_3479_MOESM1_ESM.pdf]

Supplementary file 1: Discussion questions for primary care non-rehabilitation worker feedback

- Are there any features you find confusing or difficult to use?
- Is anything missing?
- Are the instructions practical and easy to implement?
